# Typhoid vs. Remittent Fevers

**Published:** 1857-06

**Authors:** 


*Typhoid* vs. *Remittent Fevers.*—Dr. Claiborne, of Petersburg, Va., in the Stethoscope, asserts that many Virginian physicians are too hasty in acknowledging the prevalence of typhoid fever as an endemic, in that region. He still considers the form of disease under question as a remittent fever with low tendencies. We are inclined to regard this as only a repetition of the experience of the north-west. Here in Buffalo, typhoid fever was entirely unknown to the older physicians, but within a few years it has displaced the remittent form almost entirely. It is not at all surprising that in the transition stage of the epidemic constitution, the question of pathology should be gravely mooted by intelligent men. Dr. Claiborne puts out an anchor to windward in the following closing paragraphs of his interesting article:

"We do not wish to be understood as affirming that typhoid fever *never* occurs in this city or in this section of the state. On the contrary, we think we have seen undoubted cases of it. More. We have sometimes thought that there were intimations of the possibility of our malarious fevers being entirely replaced by it, in view of the fact that improvements in drainage, sewerage, &c., are gradually diminishing the causes of the former, while the increase of population, even to redundancy, in our cities, furnishes material for the enlarged development of the causes of the latter. But in none of the endemics of remittent fever, slow fever, continued fever, &c., as it is called, whether in town or country, which have come under our observation, have we been persuaded that the disease was typhoid fever proper. I have been assured by some excellent physicians of those sections of our state where the *slow fever* is almost yearly endemic, and occasionally makes devastation upan a plantation, and where it is regarded as contagious or infectious, that the disease was *undoubtedly* typhoid fever. But these same gentlemen with practical good sense have also assured me that quinine was the treatment—that they often cut short their cases with it, and that they rarely lost a patient if called in time.

"Now, while theory and experience both unite in declaring typhoid fever to be *incurable even by quinine and calomel*, these gentlemen must excuse us if we urge that they have applied to their disease a misnomer. And though we confess that it were scarcely wise to divert their attention by a new name, when they already combat their enemy so skillfully and so faithfully, yet as we advise no change in the armory, we hope they will allow us to insist on accuracy of nomenclature as essentially important to any intelligible record of the character and habits of disease."

The argument from treatment is always unsafe. It will not do to say that a disease is not typhoid fever because quinine cures it. When the change in the epidemic constitution of Virginia, which Dr. Claiborne predicts, has fairly taken place, he may find himself using quinine very freely in typhoid fever, and placing his main reliance upon it. We say this because his article is a very sensible and straight-forward production, and we believe that, as he suggests to others, he will not himself be governed by a name in the treatment of disease.

